# Imaging Features of Biliary Adenofibroma With Malignant Transformation: A Case Report and Literature Review

**DOI:** 10.7759/cureus.51575

**Published:** 2024-01-03

**Authors:** Yuanyuan Zhang, Qingqing Chen, Yue Shi, Fang Wang, Hongjie Hu

**Affiliations:** 1 School of Medicine, Shaoxing University, Shaoxing, CHN; 2 Department of Radiology, Sir Run Run Shaw Hospital, Zhejiang University, Hangzhou, CHN; 3 Department of Pathology, Sir Run Run Shaw Hospital, Zhejiang University, Hangzhou, CHN

**Keywords:** diagnosis, case report, imaging features, malignant transformation, biliary adenofibroma

## Abstract

Biliary adenofibroma (BAF) is a rare benign tumor, but it has the potential for malignant transformation. The differentiation between benign and malignant forms of BAF before surgery is of great importance for clinical decision-making. We report a case of BAF with invasive carcinoma. The patient did not present any clinical symptoms but had a history of hepatitis B virus infection for more than twenty years. Magnetic resonance imaging (MRI) revealed a solid and cystic 4 cm mass in segment II of the liver exhibiting hypointense signals on T1-weighted images and intermediate-to-high intensity signals on T2-weighted images. Enhancement scanning revealed markedly rim-like enhancement on the arterial phase, with the left inter-hepatic artery as the tumor-feeding artery, and wash-out on the venous and delayed phases. To the best of our knowledge, BAF with invasive carcinoma is uncommon. Preoperative qualitative diagnosis based on imaging features can achieve the maximum benefit for patients.

## Introduction

The benign bile duct tumors include biliary adenofibroma (BAF), bile duct adenoma, bile duct hamartoma (von Meyenburg complexes), biliary cystadenoma, etc. Among them, BAF is an extremely rare solid-cystic tumor, which was first described by Tsui et al. in 1993 [1]. It has the characteristics of a slow-growing benign tumor but with the potential for malignant transformation. In this article, we report a rare case of BAF previously misdiagnosed as hepatocellular carcinoma (HCC) and review the relevant literature to explore its clinical symptoms, imaging findings, as well as differential diagnosis and prognosis to improve the understanding of the disease.

## Case presentation

A 71-year-old woman accidentally found a left hepatic space-occupying lesion during a routine physical examination in our hospital. The patient did not present any clinical symptoms but had a history of hepatitis B virus infection for more than 20 years. Laboratory examination showed liver dysfunction and low platelets, the AST and ALT were 70 U/L (13-35 U/L) and 74 U/L (7-40 U/L), respectively, and the platelet count was 107 × 10^-9^/L (125-350 × 10^-9^/L). Tumor markers such as AFP (0.0-40.0 ng/ml) and CA19-9 (0.0-5.0 ng/ml) were not elevated. Neither the patient nor his family had any history of liver-related surgery.

**Figure 1:**
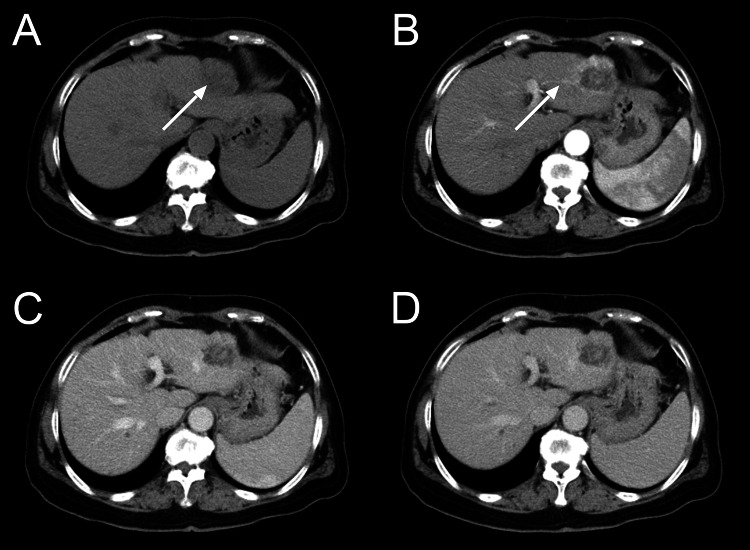
CT imaging of BAF. (A) An abdominal plain CT scan revealed a subcapsular solid-cystic mass with an obscure boundary (white arrow) in the liver segment II. The mass showed markedly heterogeneous enhancement in the (B) arterial phase, followed by wash-out in the (C) venous and (D) delayed phases. The arterial phase (B) also showed that the blood supply came from the left inter-hepatic artery and its branches. BAF: Biliary adenofibroma.

Contrast-enhanced CT revealed a low-density mass of 4 cm × 4 cm in segment II of the liver, which showed heterogeneous enhancement on the arterial phase and wash-out on the portal venous and delayed phase (Figure 1). According to the imaging features of CT, HCC is the first consideration in clinical diagnosis and needs to be differentiated from cholangiocarcinoma and liver metastases.

**Figure 2:**
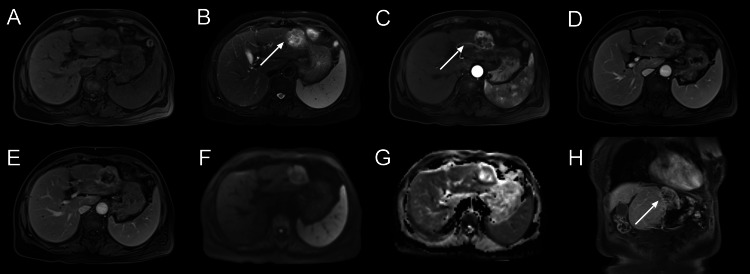
MRI imaging of BAF. (A) T1-weighted image. (B) T2-weighted image. Contrast-enhanced MRI demonstrated markedly heterogeneous enhancement in the (C) arterial phase of the lesion but low signal in the (D) venous and (E) delayed phases. Diffusion and apparent diffusion coefficient (ADC) images (F and G) showed restricted diffusion. (H) Coronal (white arrow). The mass (B) had an obscure border (white arrow). The (C) arterial phase showed that the blood supply came from the left inter-hepatic artery and its branches (white arrow). BAF: Biliary adenofibroma.

MRI has better soft tissue resolution and allows for a more accurate qualitative diagnosis of liver masses. For further examination, an abdominal MRI was performed, which exhibited a heterogeneously mixed-signal mass with unclear boundaries. Enhancement scanning revealed markedly rim-like enhancement on the arterial phase, with the left inter-hepatic artery as the tumor-feeding artery, and wash-out on the venous and delayed phases. Diffusion-weighted imaging (DWI) sequence showed moderate hyperintensity, and the apparent diffusion coefficient (ADC) coefficient value was 1.260 × 10^-3 ^mm^2^/s, which indicated that there was an obvious limitation to diffusion in the solid portion (Figure 2). The imaging findings were similar to hepatocellular carcinoma (HCC), and liver metastases need to be identified. Understanding the medical history of primary malignant tumors helps make a correct diagnosis.

**Figure 3:**
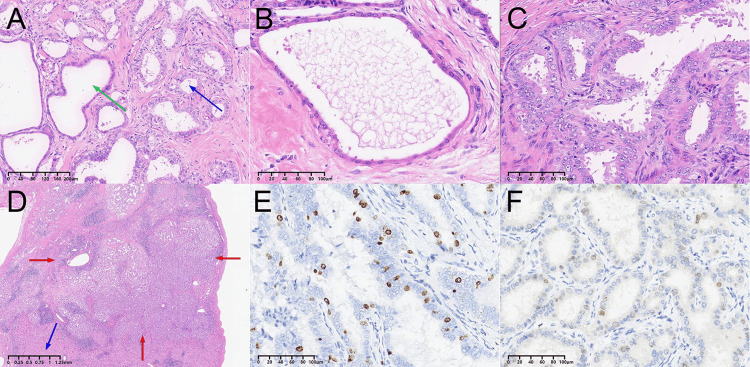
(A) The tumor was composed of tubuloglandular and microcytic structures embedded in a fibrous matrix. The green arrow shows the microcystic structures. The blue arrow shows the tubuloglandular structures (H&E, 100×). (B) The tumor areas were lined with cuboidal or low-columnar biliary epithelium. Nuclei were round or oval with small nucleoli, and cytoplasm showed without atypia (H&E, 200×). (C) In some areas, papillary projections were present in the lumens. The epithelial showed mild to severe atypical hyperplasia with columnar-type epithelium with elongated, hyperchromatic nuclei and nuclear crowding (H&E, 200×). (D) There was invasive growth in the adjacent parenchyma of the liver. The blue arrow shows normal liver tissue. The red arrow shows invasive growth tissue (H&E, 10×). (E) The malignant part of the tumor had an increased Ki-67 index (20%) (immunostaining, 200×). (F) p53 was negative (immunostaining, 200×).

The patient underwent a left lateral hepatectomy in May 2022. According to intraoperative findings, there was a hard and exophytic mass under the left liver capsule with irregular margins. The final histopathological diagnosis was BAF with invasive carcinoma (Figure 3). The patient was discharged from the hospital three days after surgery, and no signs of recurrence were found at 12 and 18 months of follow-up.

## Discussion

BAF is considered a benign and complicated tubulocystic tumor with abundant fibrous stroma [1]. It is considered a benign and complicated tubulocystic tumor with abundant fibrous stroma, but some of them have the possibility of malignant transformation, including atypical hyperplasia, invasive cancer, or distant metastases. To date, 34 BAF cases have been reported. Among them, 15 cases were benign, and 19 cases were accompanied by malignant transformation. The case presented in our hospital was BAF with invasive carcinoma. We analyzed the pathological data and found that two of the 18 cases of malignant BAF had metastases during the follow-up [2,3]. Two of the 16 cases of benign BAF had local recurrence during follow-up [4]. We made a detailed analysis of reported cases and found that the clinical presentation and laboratory examination of BAF are nonspecific. These cases consisted of 16 males and 19 females. The median age was 63 and ranged from 23 to 83 years. More than half of the patients presented with epigastric pain, and 30% had no clinical symptoms. Three cases had an elevated CA19-9 concentration [3,5,6], and one case had an elevated CA12-5 concentration [7]. The other three cases had hepatitis B [8-10], and one case had hepatitis C [11]. Therefore, the image characteristics improve the accuracy of preoperative diagnosis and evaluation of benign and malignant lesions of BAF before operation, which is important for clinical decision-making.

We performed a detailed analysis of imaging information from previous cases (see Appendix) in which the median size of the lesion was 6.55 and ranged from 0.7 to 20 cm, which was mainly located under the capsule (50%) [1-20]. Most of the lesions were multilobulated and multiseptated cystic. Contrast enhancement showed that the separation was obviously enhanced. Some lesions have obvious solid components, accompanied by enhanced arterial phase enhancement, of which nine cases described relatively low signal on the venous and delay phases. The pathological diagnosis showed malignant transformation in seven cases [3,6,10,11,13,16,17], which may be related to complex papillary, cribriform-like, back-to-back architecture, and less fibrous tissue. Delayed enhancement of benign BAF may be related to a high content of fibrous stroma [1]. Among the cases that mentioned the DWI sequence, five showed obvious diffusion restriction, and the pathology suggested malignant transformation [6,11,13,16,17]. In summary, a lobulated and multiseptated cystic-solid mass located under the hepatic capsule, with the separation obviously enhanced, can help radiologists associate BAF. However, these imaging features are not specific to BAF and need to be diagnosed by histopathology. The following features may suggest malignant BAF: (1) prominent solid components with cystic elements, (2) unclear border, (3) obvious enhancement in the arterial phase followed by wash-out in the venous and delayed phase, and (4) restricted diffusion. In addition, BAF should be distinguished from other liver lesions containing cystic components to avoid misdiagnosis.

Congenital conditions

Caroli disease

Caroli disease is characterized by congenital dilatation of bile ducts. The lesion showed different degrees of dilatation of the intrahepatic bile duct around the small vessels, and the central enhancement could be seen after enhancement.

von Meyenburg complexes

von Meyenburg complexes (VMCs) are considered congenital bile duct malformations due to the failure of embryonic involution, which often occurs in multiples, are less than 5 mm in diameter, are well-circumscribed, and have no enhancement after enhanced scanning.

Acquired inflammatory conditions

Liver abscess

Liver abscesses often present as thick-walled cysts with perilesional edema, and the liquid-gas level is a characteristic manifestation. Regarding neoplastic conditions, HCC with necrosis usually occurs in patients with cirrhosis, and the elevated serum AFP can aid in a correct diagnosis.

Cystic metastases

Lesions usually appear as multiple, round, unilocular cysts, and definite primary tumors can help to diagnose.

Intraductal papillary neoplasms of the bile duct

It is known that intraductal papillary neoplasms of the bile duct (IPNB) are characterized by visible intraductal mass in the dilatation of the intrahepatic or extrahepatic bile ducts, and the up and downstream bile duct can also be dilated.

Mucinous cystic neoplasm

Most mucinous cystic neoplasm (MCN) cases occur in women with ovarian-like stroma. MCNs appear to be cystic tumors with irregular walls and internal septations.

Histopathological examination of the tumor is the gold standard for the diagnosis of BAF. Reviewing the published cases and imaging findings, although these mentioned imaging features are nonspecific, CT and MRI are helpful in the diagnosis of BAF and the evaluation of preoperative invasiveness. The imaging features of solitary, clear boundary, less solid components, and delayed enhancement suggest that there is no malignant transformation of BAF. Multiple lesions, unclear borders, more solid components, obvious enhancement of solid components in the arterial phase with the patterns of wash-in and wash-out on portal venous and delayed phases, and the restricted diffusion on the DWI phase can help us diagnose malignant BAF. In addition, further investigations on the role of advanced imaging techniques, such as contrast-enhanced ultrasound (CEUS), dual-energy computed tomography (DECT), and positron emission computed tomography (PET-CT), in characterizing BAF are required.

## Conclusions

As a result, BAF is a rare benign liver tumor with highly malignant transformation potential. This case showed a lobulated and multiseptated cystic-solid mass under the capsule of the left liver with enhanced fine separation, which is evidence for the diagnosis of BAF. The obscure margins of the lesion, obvious enhancement of solid components in the arterial phase with the patterns of wash-in and wash-out on portal venous and delayed phases, and the restricted diffusion on the DWI phase can help us diagnose malignant BAF. Surgical management is considered as the main treatment modality. We hope that this case serves to highlight the signs of malignant transformation of BAF and assist in surgical and clinical decision-making.

## Appendices

**Table 1:** Literature review and analysis of pathological, clinical, and radiological data AFP: Alpha-fetoprotein; CA: Carbohydrate-antigen; MRI: Magnetic resonance imaging; CT: Computed tomography; US: Ultrasound; CK: Cytokeratin; CEA: Carcinoembryonic antigen; SMA: Smooth muscle actin; CDKN2A: Cyclin-dependent kinase inhibitor; CCND1: CyclinD1; ERBB2: Her2/neu gene.

Author/Year	Sex/age (years)	Symptoms	Laboratory workup	Image examination	Location	Size (cm)	Malignant transformation	Immunohistochemistry/molecular studies	Follow-up
Akin et al. [2]/2002	Male/25	Abdominal enlargement, right upper quadrant pain	Normal	CT: Low-density mass with an obscure margin. Contrast enhancement was heterogeneous throughout the mass and necrosis in the center	Subcapsular area of the right lobe	20	Yes	/	Recurrence and pulmonary metastasis after 3 years
Garduño-López et al. [5]/2002	Female/68	Right upper quadrant pain, vomiting, diarrhea, and jaundice	Elevated CA19-(965.8U/ml)	US: Hypoechoic mass, CT: Low-density mass	Subcapsular area of the left lobe	6	No	Positive: Stained CA 19-9	No recurrent after 30 months
Varnholt et al. [12]/2003	Female/47	Right upper quadrant pain	Normal	US: Hyperechoic mass. CT: Solid-cystic mass and low-density mass. MRI: Lobulated and multiseptated solid-cystic mass. Hypointensity on T1WI and hyperintensity on T2WI with fine separation. Contrast enhancement showed that the solid composition and separation were obviously enhanced.	Subcapsular area of the left lobe	16	No	Positive: Epithelial: D10, P53, Cam5.2/AE1/3, CK7, CK19, CEA; EMA Stroma: vimentin and SMA	Partial resection of the tumor. No metastasis or significant growth after 3 years
Kai et al. [8]/2012	Male/40	Upper abdominal pain	Carrier for HBV	CT: Lobulated and multiseptated cystic mass	Subcapsular area of the right lobe	7	Yes	Positive: CK19, CA 19-9, MUC 1	No recurrence. Dying of fulminant hepatitis B 8 months after surgery.
Tsutsui et al. [13]/2014	Female/69	Asymptomatic	Normal	US: Hyperechoic mass. CT: Lobulated solid-cystic mass with fine separation. MRI: Lobulated solid-cystic mass. Low and high confounding signals on T1WI and T2WI. Contrast enhancement showed that the solid composition and separation were obviously enhanced in the arterial phase and relatively low signal in the venous and hepatobiliary phases. Restricted diffusion of the lesion.	Subcapsular area of the right lobe	3.5	Yes	Positive: CK7, CK19, CAM5.2, CKAE1/AE3 and EMA	No recurrent after 4 years
Jacobs et al. [14]/2015	Female/57	Left lower quadrant pain	Normal	CT: Lobulated low-density mass with fine separation. Contrast enhancement demonstrated markedly heterogeneous enhancement in the lesion.	Subcapsular area of the right lobe, extending into the segment of the left hepatic lobe	11.8	Yes	/	No recurrence after 5 years
Thompson et al. [16]/2016	Male/71	Asymptomatic	Normal	US: A mass. MRI: Lobulated and multiseptated solid-cystic mass. Low and high confounding signals on T1WI and T2WI. Contrast enhancement showed that wash-in the arterial phase followed by wash-out in the venous phase.	Subcapsular area of the left lobe	9	Yes	/	No recurrence. Dying for primary lung malignancy after 9 years.
Thompson et al. [16]/2016	Male/71	Asymptomatic	Normal	MRI: Lobulated and multiseptated cystic mass. Hypointensity on T1WI and hyperintensity on T2WI. Contrast enhancement demonstrated rim enhancement in the delay phase. Restricted diffusion of the lesion.	Caudate lobe	6.6	Yes	Positive: CK7, CDKN2A mutation	No recurrence after 1 month
Kaminsky et al. [19]/2017	Female/37	Postprandial nausea, vomiting, and upper quadrant pain	Normal	CT: Multilocular cystic mass. MRI: Lobulated solid-cystic mass. Low and high confounding signals on T1WI and T2WI. Contrast enhancement demonstrated rim enhancement.	Right hepatic lobe	4.5	Yes	Positive: Ki-67, CK7, CK19, CD56	No recurrence after 4 months
Chua et al. [11]/2018	Female/66	Asymptomatic	Carrier for HCV	MRI: Contrast enhancement showed that the lesion was obviously enhanced in the arterial phase and relatively low signal in the venous and delay phases. False capsules can be seen in the mass. Restricted diffusion of the lesion.	Liver segment IV b	6	Yes	Positive: CK7, P53	No recurrence after 6 weeks
Lee et al. [9]/2019	Male/63	Asymptomatic	Chronic hepatitis B	CT: A slow-growing low-density mass. MRI: Multilocular cystic mass with fine separation. Hypointensity on T1WI and hyperintensity on T2WI. Contrast enhancement showed that the separation was obviously enhanced.	Subcapsular of the IV and VIII segments	4.5	No	Positive: CK7, CK19, P53 (focally positive), SMA	No recurrence after 41 months
Lee et al. [9]/2019	Male/38	Asymptomatic	Normal	CT: Low-density mass. MRI: Lobulated and multiseptated cystic mass. Hypointensity on T1WI and hyperintensity on T2WI. Contrast enhancement showed that the separation was obviously enhanced.	Subcapsular area of the left lobe	2.5	No	/	No recurrence after 39 months
Hu et al. [10]/2022	Female/64	Right upper quadrant pain	Carrier for HBV	US: Hypoechoic nodules. CT: Low-density solid-cystic mass. Contrast enhancement showed that wash-in the arterial phase followed by wash-out in the venous phase.	Subcapsular area of the right lobe	1.8	Yes	Positive: CK7, CK19, CEA, KI67 (less than 10% in benign part; 20%–30% in malignant part)	No recurrence after 9 months
Li et al. [20]/2022	Male/68	Abdominal pain	Normal	US: Hyperechoic nodules. After contrast injection, the lesion enhanced. CT: Low-density mass. MRI: Cystic mass, hypointensity on T1WI, and hyperintensity on T2WI. Enhanced MRI scan showed obvious enhancement of solid components and separation of lesions in the arterial phase, the degree of lesion enhancement decreased in the portal phase and the hepatobiliary phase, and enhancement of the bile duct structure was found in the lesion.	Subcapsular area of the left lobe	/	No	/	No recurrence after 1 month
Liu et al. [6]/2022	Female/30	Asymptomatic	Elevated CA19-9 (42.5 U/mL)	CT: Low-density mass. Contrast enhancement showed that the solid composition and separation were obviously enhanced in the arterial phase and relatively low signal in the venous phases. MRI: Solid-cystic lobulated mass. Hypointensity on T1WI and hyperintensity on T2WI. Contrast enhancement showed that the solid composition was obviously enhanced. Restricted diffusion of the lesion. CTA showed that the blood supply came from the left inter-hepatic artery.	Subcapsular area of the left lobe	8.5	Yes	Positive: vimentin (+), TFE-3 (+), FLi-1 (+), actin minority (+), S-100 minority (+), P53 about 40% (+), Ki67 hot spot area about 10% (+), SMMHC part (+), CK part (+), CK7 minority (+), CK8/18 minority (+), P63 minority (+), B-catenin (+)	No recurrence after 6 months
Alshbib et al. [3]/2022	Male/63	Upper abdominal pain	Elevated CA19-9 (163 U/mL)	CT: Multilocular solid mass. Contrast enhancement showed that the lesion was obviously enhanced in the arterial phase and relatively low signal in the venous phase. MRI: Low and high confounding signals on T1WI and T2WI. The mass was with focal hemorrhage. Contrast enhancement demonstrated rim enhancement.	Subcapsular of the IV b and V segment	15.5	Yes	Positive: CK7, CK20, MUC1, CEA, CD56, Smad4, BAP1, CCND1	Metastasis occurred after 3 months
Wang et al. [17]/2022	Female/51	Upper abdominal pain	Elevated CRP (75.1 mg/L)	CT: Solitary mass. Contrast enhancement showed that the lesion was obviously enhanced in the arterial phase and relatively low signal in the venous phase. MRI: Solid-cystic mass. Hypointensity on T1WI and hyperintensity on T2WI. Restricted diffusion of the lesion.	Subcapsular area of the right lobe	9.2	Yes	Positive: CK7, CK19	No recurrence after 12 months
Kang et al. [18]/2023	Male/64	Asymptomatic	Normal	MRI: Lobulated and multiseptated cystic mass. Hypointensity on T1WI and hyperintensity on T2WI. Contrast enhancement showed that the solid composition and separation were obviously enhanced in the arterial phase and relatively low signal in the venous and hepatobiliary phases.	Subcapsular area of the right lobe	9.1	/	Positive: CK7, CK19, MUC-1, EMA, P53, Ki-67	/

## References

[1] Tsui WM, Loo KT, Chow LT, Tse CC (1993). Biliary adenofibroma. A heretofore unrecognized benign biliary tumor of the liver. Am J Surg Pathol.

[2] Akin O, Coskun M (2002). Biliary adenofibroma with malignant transformation and pulmonary metastases: CT findings. AJR Am J Roentgenol.

[3] Alshbib A, Grzyb K, Syversveen T, Reims HM, Lassen K, Yaqub S (2022). Biliary adenofibroma: a rare liver tumor with transition to invasive carcinoma. Case Rep Surg.

[4] Arnason T, Borger DR, Corless C (2017). Biliary adenofibroma of liver: morphology, tumor genetics, and outcomes in 6 cases. Am J Surg Pathol.

[5] Garduño-López AL, Mondragón-Sánchez R, Bernal-Maldonado R (2002). A case of biliary adenofibroma of the liver causing elevated serum CA 19-9 levels. Rev Oncol.

[6] Liu SR, Zhu Q, Feng MB, Yan Q, Zhu TF, Xu LB (2022). Atypical biliary adenofibroma of the liver: related treatment and performance. Case Rep Gastroenterol.

[7] Nguyen NT, Harring TR, Holley L, Goss JA, O'Mahony CA (2012). Biliary adenofibroma with carcinoma in situ: a rare case report. Case Reports Hepatol.

[8] Kai K, Yakabe T, Kohya N (2012). A case of unclassified multicystic biliary tumor with biliary adenofibroma features. Pathol Int.

[9] Lee S, Kim KW, Jeong WK, Yu E, Jang KT (2019). Magnetic resonance imaging findings of biliary adenofibroma. Korean J Gastroenterol.

[10] Hu W, Zhao Y, Liu Y, Hua Z, Liu A (2022). Imaging features of biliary adenofibroma of the liver with malignant transformation: a case report with literature review. BMC Med Imaging.

[11] Chua D, Chiow AK, Ang TL, Wang LM (2018). Malignant transformation arising within unusual and rare hepatic lesions: fibropolycystic disease form of ductal plate malformation and biliary adenofibroma. Int J Surg Pathol.

[12] Varnholt H, Vauthey JN, Cin PD, Rde WM, Bhathal PS, Hughes NR, Lauwers GY (2003). Biliary adenofibroma: a rare neoplasm of bile duct origin with an indolent behavior. Am J Surg Pathol.

[13] Tsutsui A, Bando Y, Sato Y (2014). Biliary adenofibroma with ominous features of imminent malignant changes. Clin J Gastroenterol.

[14] Jacobs MA, Lanciault C, Weinstein S (2015). Incidental biliary adenofibroma with dysplastic features. BJR Case Rep.

[15] Godambe A, Brunt EM, Fulling KH, Reza Kermanshahi T (2016). Biliary adenofibroma with invasive carcinoma: case report and review of the literature. Case Rep Pathol.

[16] Thompson SM, Zendejas-Mummert B, Hartgers ML, Venkatesh SK, Smyrk TC, Mahipal A, Smoot RL (2016). Malignant transformation of biliary adenofibroma: a rare biliary cystic tumor. J Gastrointest Oncol.

[17] Wang SC, Chen YY, Cheng F, Wang HY, Wu FS, Teng LS (2022). Malignant transformation of biliary adenofibroma combined with benign lymphadenopathy mimicking advanced liver carcinoma: a case report. World J Clin Cases.

[18] Kang Y, Wang W, Zhang Y (2023). MRI imaging features of the biliary adenofibroma. Asian J Surg.

[19] Kaminsky P, Preiss J, Sasatomi E, Gerber DA (2017). Biliary adenofibroma: a rare hepatic lesion with malignant features. Hepatology.

[20] Li SP, Wang P, Deng KX (2022). Imaging presentation of biliary adenofibroma: a case report. World J Clin Cases.

